# Chernobyl at 40 years: leukaemias, cancers and implications for nuclear safety

**DOI:** 10.1038/s41375-026-03027-9

**Published:** 2026-08-24

**Authors:** RP Gale

**Affiliations:** Centre for Haematology, Department of Immunology and Inflammation, Imperial College of Science, Technology and Medicine, London, UK

**Keywords:** Cancer epidemiology, Health care

40 years ago, on April 28, 1986, I received a 3 AM call from Anatoly Dubrinin, the Soviet Union Ambassador to the US in Washington. He told me President Mikhail Gorbachev wanted me to come to Moscow immediately. I guessed it was about the Chernobyl nuclear power facility accident 2 days earlier, details of which were hidden by Soviet authorities but revealed soon thereafter when the radioactive cloud released from the reactor complex near Kyiv, Ukraine reached radiation sensors at a Swedish nuclear power facility. I knew Mr. Gorbachev from meeting a few years before at the funeral of his mentor, General Secretary Yuri Andropov who led the USSR briefly. I offered help to treat the most seriously injured radiation victims.

My colleagues and I on our joint US (Richard Champlin, Paul Terasaki), Israel (Yair Reisner) and, Soviet team, reported results of treating victims with acute radiation syndrome (>2 Gy). We were mostly successful with only 29 deaths amongst more than 200 people exposed to high radiation doses [1]. 3 others were killed by the explosion at the reactor complex; 32 immediate deaths. This was the 1st use of a molecularly-cloned myeloid haematopoietic growth factor in humans [2].

With a 40-year follow-up, it’s time to assess the long-term health effects of the world’s worst nuclear accident and consider implications for the recent growth of nuclear facilities to help reverse global warming and the spread of nuclear weapons.

People reading popular news sources or even some scientific reports will surely be confused about Chernobyl’s long-term health consequences. Some sources report thousands, hundreds of thousands or even millions of cancers caused by radiation released from Chernobyl. Other sources claim few, if any, additional cancers. There are other reports claiming many birth defects, congenital abnormalities and even heritable genetic mutations. Which of these claims is true?

Much of what we know about radiation and cancer comes from a studies of people exposed to the A-bomb at Hiroshima and Nagasaki and other Japanese not in these cities when the bombs exploded. What do these data show? First, the risk of developing a radiation-related cancer is dose-dependent; the higher the dose, the greater the risk of developing cancer. Second, radiation-related leukaemias occurred within a few years, whereas other cancers occurred only after several decades. Conclusions drawn from studies of the A-bomb survivors are mostly confirmed by studies in other radiation-exposed populations. However, radiation exposures from Chernobyl are different than those of the A-bomb survivors. For example, radiation exposure in Japan was instantaneous whereas population exposures from Chernobyl occur over decades in most instances. As such, one must be cautious when applying estimates from the A-bomb survivors to persons exposed to radiation from the Chernobyl nuclear power facility accident.

Most scientists accept the linear no threshold model of radiation exposure and cancer risk impling every excess radiation exposure, no matter how small, increases a person’s cancer risk. Some people dispute this model, with some even claiming excess radiation doses are beneficial (hormesis). Few data support this notion (Reviewed in [3]).

Let’s consider the impact of radiation exposures from the Chernobyl accident on the 6.6 billion people living in the Northern Hemisphere including the 200 million people living or who will live in Ukraine, Belarus and Russia. For example, the average dose from the Chernobyl accident to the about 300,000 evacuees and 1 million people living in contaminated lands is equivalent to the background radiation dose received from living 3 to 10 years in Los Angeles. Based on calculations like these, the UN Scientific Committee on the Effects of Atomic Radiation (UNSCEAR) predicted about 4000 excess cancers over the first 50 years. The Chernobyl Forum predicted 10,000 excess cancer deaths. Other sources, often without scientific credentials or with a political agenda, predict thousands, hundreds of thousands or even millions of excess cancers and cancer deaths. (Reviewed in [4];).

Putting aside these predictions, which are fundamentally unverifiable, what do the data show? First, there were about 7000 excess thyroid cancers in children and adolescents living near the accident site, an estimated 100-fold increased incidence. Tragic, but fortunately < 10 deaths. These cancers were caused by exposure to radioactive ^131^I in contaminated milk. There is one report of increased chronic lymphocytic leukaemia and another of increased breast cancer [5, 6]. Neither is convincing.

The bottom line is that a few convincing data show radiation released from Chernobyl increased cancers globally. Admittedly, failing to detect increased cancers does not prove that cancers were not increased. However, any increase must be relatively small and below our level of detection. Recall that about 40 percent of us will develop cancer in our lifetime. Even if there were an increase from Chernobyl-related radiation, it would be a very small percentage of the normal cancer rate over 70 years since the accident, diluted in about 4 billion cancers. Importantly, there are also no convincing data that birth defects or genetic abnormalities were increased by the radiation released from Chernobyl despite what you might read elsewhere. Predicted cancers from the Fukushima-Daichi nuclear power facility in Japan in 2011 are few, if any [7].

Next, we need to put electricity production from nuclear energy in the context of other energy alternatives and of global climate change. There are more than 400 nuclear power reactors operating worldwide, providing about 10 percent of global energy, with >50 under construction. Most people understand that burning fossil fuels worsens global warming, but not that it releases more radiation into the environment *per* kilowatt generated compared with a nuclear power facility. (Coal contains radionuclides released upon burning.) An estimated 10,000 people die each year globally from coal mining. Mining copper pipes for a solar power station also brings more radiation to the earth’s surface than radiation from a nuclear power facility. Photovoltaic solar energy results in toxic chemicals, which, unlike radioisotopes, never decay. The same for electrical storage batteries. As for cancer, consider that the construction of the Aswan High Dam in Egypt caused many thousands of bladder cancers caused by Schistosomiasis and blindness from glaucoma because of the slowed flow of the Nile. There are no simple answers.

The greatest dangers associated with recent efforts to expand commercial nuclear power generation are not accidental radiation releases. Safer reactor designs are available and the challenge of spent nuclear fuel disposal is soluble absent politics. The greatest dangers are using ‘civilian’ reactors to produce weapons-grade materials and reactors as military targets. For example, the Chernobyl reactor lacked a containment building because its large size and graphite moderator allowed production of weapons-grade plutonium.

There are several examples of attacks on nuclear power facilities, including in Syria and Iraq, but most of these occurred before they contained nuclear fuel. However, after the invasion of Ukraine, the Russian Federation Army hijacked the Zaporizhia nuclear power facility, the largest in Europe and turned it into a military camp. Recently, a Russian drone struck and damaged a modern containment facility protecting the entombed Chernobyl nuclear power facility. For several weeks, the US and Israel have been bombing Iranian nuclear facilities which they consider miliary targets. A few weeks ago, there was an attack near the Bushehr commercial nuclear power facility in southern Iran. The latest is an Iranian drone attack near the UAE’s Barakah nuclear power facility. These actions fall somewhere between dangerous and crazy.

Attacks against civilian nuclear facilities are prohibited by several international conventions to which the US is a signatory unless the facility supports miliary purposes. There is no evidence that this is so for any of these civilian facilities. These attacks could easily escalate with Iran attacking the Dimona nuclear weapons facility in Israel and attacks against nuclear facilities in Europe and the US [8]. In places like Israel and the Middle East, large populations might need to be evacuated and large amounts of land quarantined.

In summary, the Chernobyl nuclear power facility accident was a global tragedy. Although I focused on leukaemias and cancer, we should not forget the important social, psychological and economic consequences to exposed populations. The Chernobyl accident was preventable, resulting from human error and faulty reactor design. We now have safer nuclear reactors and have learned important lessons from the Chernobyl and Fukushima nuclear power facility accidents. It’s time for the US to re-evaluate the potential role of nuclear energy in helping us deal with global climate change. And we need to strengthen international treaties and protocols to prevent attacks on civilian nuclear facilities and try to curtail the spread of nuclear weapons.
